# Intervention effects in the transmission of COVID-19 depending on the detection rate and extent of isolation

**DOI:** 10.4178/epih.e2020045

**Published:** 2020-06-23

**Authors:** Okyu Kwon, Woo-Sik Son, Jin Yong Kim, Jong-Hun Kim

**Affiliations:** 1 National Institutes for Mathematical Sciences, Daejeon, Korea; 2 Division of Infectious Diseases, Department of Internal Medicine, Incheon Medical Center, Incheon, Korea; 3 Department of Social and Preventive Medicine, Sungkyunkwan University School of Medicine, Suwon, Korea

**Keywords:** COVID-19, Agent-based model, Non-pharmaceutical intervention, Social network, Isolation

## Abstract

**Objectives:**

In 2020, the coronavirus disease 2019 (COVID-19) respiratory infection is spreading in Korea. In order to prevent the spread of an infectious disease, infected people must be quickly identified and isolated, and contact with the infected must be blocked early. This study attempted to verify the intervention effects on the spread of an infectious disease by using these measures in a mathematical model.

**Methods:**

We used the susceptible-infectious-recovery (SIR) model for a virtual population group connected by a special structured network. In the model, the infected state (*I*) was divided into *I* in which the infection is undetected and *I_x_* in which the infection is detected. The probability of transitioning from an I state to *I_x_* can be viewed as the rate at which an infected person is found. We assumed that only those connected to each other in the network can cause infection. In addition, this study attempted to evaluate the effects of isolation by temporarily removing the connection among these people.

**Results:**

In Scenario 1, only the infected are isolated; in Scenario 2, those who are connected to an infected person and are also found to be infected are isolated as well. In Scenario 3, everyone connected to an infected person are isolated. In Scenario 3, it was possible to effectively suppress the infectious disease even with a relatively slow rate of diagnosis and relatively high infection rate.

**Conclusions:**

During the epidemic, quick identification of the infected is helpful. In addition, it was possible to quantitatively show through a simulation evaluation that the management of infected individuals as well as those who are connected greatly helped to suppress the spread of infectious diseases.

## INTRODUCTION

The World Health Organization (WHO) declared a pandemic on March 11, 2020 for a new viral infection (coronavirus disease 2019, COVID-19) first reported in China in November 2019 [1]. Since treatments and vaccines are not readily available for a new infectious disease, especially during a global pandemic, using non-pharmaceutical interventions to minimize the peak of infection associated with the outbreak and enforcing a “mitigation strategy” so as not to overload the healthcare system is the only feasible response [2]. The most intuitive and effective method among non-pharmaceutical interventions is to “diagnosis and isolate infected people as soon as possible” [3,4]. Using a mathematical model, this study aimed to determine how quick detection of COVID-19 and isolation of infected individuals can help prevent the transmission of infectious diseases.

## MATERIALS AND METHODS

### Model

**Figure 1.**
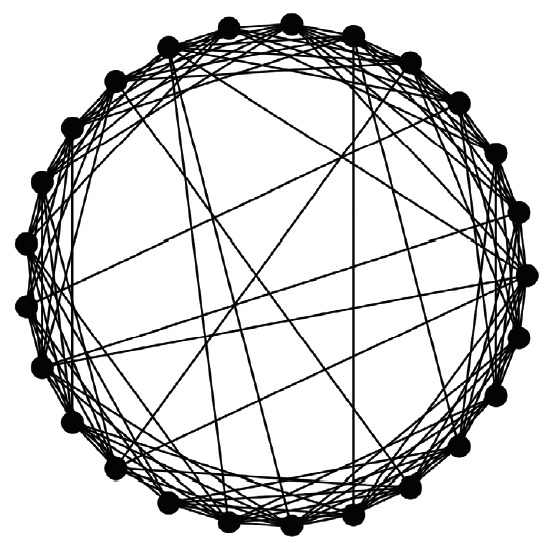
Concept diagram of the small-world network proposed by Watts & Strogatz [5]. Each dot represents a person, and the line between dots indicates a connection between them. Contact between two people is possible only between two points where a connecting line exists. This figure shows an example in which 25 people are connected for visual convenience.

**Figure 2.**
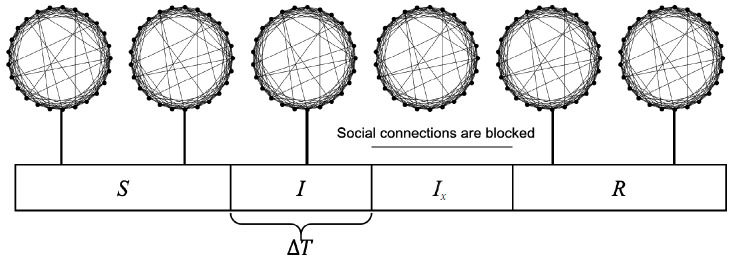
The process by which a person’s social connections change over time. In the susceptible state (*S*), infection may be caused by a nearby infected neighbor. An infected state (*I*) can cause infection for a period of time (*∆T*) in surrounding people who are connected. After *∆T* in state *I*, the infection becomes detected (*I_x_*). In state *I_x_*, social connections are blocked and infection cannot spread to surrounding individuals. When state *R* is reached after recovery from the infection, the infection can no longer be transmitted even if the social connection is restored.

**Figure 3.**
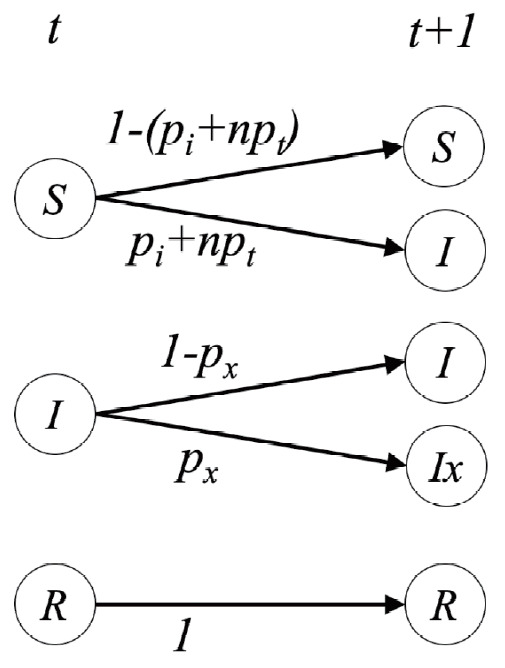
Stochastic changes in the susceptible-infection-recovery (SIR) model over time. The state at time *t* is stochastically changed to the state at time *t*+1. After the initial infection, whether it remains infectious (*I*) or is detected (*I_x_*), the recovery state (*R*) is reached unconditionally after 21 days (3 weeks). *p_i_* is the probability of the occurrence of primary infection. *np_t_* is the probability that n infected people in state I connected to those in state *S* can spread the infection. *p_x_* is the probability that an infected person in state *I* will be confirmed for the infection.

A virtual population group was assumed where 10,000 people were connected by a specific network. It was assumed that contact was made only between people who are connected by a link. Each person is connected to an average of 10 others. We introduced a small-world network proposed by Watts & Strogatz [5], where 10% of all connections are randomly distant connections (Figure 1). It was assumed that the connection structure between people did not change with time. Many existing studies have examined a model which calculates the spread of an infectious disease using a network that is a set of nodes and links, where the nodes represent each individual and the links represent infection channels between people [6-8]. We used the most basic susceptible-infectious-recovery (SIR) model as a disease simulation model [9,10]. However, the infected state (*I*) was left as if undetected and was changed to *I_x_* if detected (Figure 2). In reality, an infected person cannot immediately recognize the fact that he/she is infected. Also, when the infection is undetected, the infected person continues daily activities transmitting the infection to others. In contrast, those who recognize their infection no longer spread the infection after being treated at the hospital. Therefore, it was assumed that those who are infected but have been categorized as *I_x_* after recognizing their infection would no longer spread the infection to others around them. Only those who have not recognized their infection can infect those who are connected to them with a certain probability (*p_t_*) (Figure 3). There is a probability, *p_x_*, of changing from an infected state (*I*) to a state in which the infection is detected (*I_x_*). The sum of the duration of the *I* and *I_x_* states was set to 3 weeks in total (21 days). Therefore, if the *p_x_* value is sufficiently small, a person could stay in the *I* state for 3 weeks before reaching the recovery (*R*) state. The *p_i_* is the probability of generating the initial infected case. This is likely to be a naturally occurring infection or infection from outside the target population, and this value is set to a small size. As such, there are three parameters in this model, including *p_t_*, *p_x_*, and *p_i_*.

The stochastic process in which a person’s disease state changes over time is shown in Figure 3, and the calculation was made by setting the time interval to an hour. A person in the susceptible (*S*) state at time *t* can change to state *I* at time *t*+1 with a probability of *p_i_*. If a person in state *S* at time *t* has *n* number of infected people who are connected to them, the person could change to state *I* at *t*+1 with a probability of *n*×*p_t_*. A person in state *I* at time *t* can either change to state *I_x_* at time *t*+1 with a probability of *p_x_* or stay in state *I* with a probability of 1-*p_x_*. The person in state *I* at time *t* always enters the state of recovery (*R*) at time *t*+24×21 (3 weeks), and those who reach state *R* maintain state *R* from then on.

### Ethics statement

This study is about a theoretical mathematical model that evaluates the intervention effect of infectious disease spread. Consent to use personal information is not a subject to be considered.

## RESULTS

Three scenarios were simulated to verify the effectiveness of rapid infection detection and isolation. In the first scenario, social connections were blocked only for individuals whose infection was detected. In the second scenario, if an infection was confirmed among those who are directly connected to someone whose infection was detected, the infection is immediately changed to detected and social connections were blocked. It simulates a situation in which an infection is quickly identified through epidemiological investigations of those who have come into contact with an infected person. In the third scenario, among those who are linked to the confirmed infector (*I_x_*), the infected person (*I*) becomes the confirmed state (*I_x_*) and the non-infected person is quarantined for 14 days. This scenario minimizes social contact, even for non-infected people, because there is a relatively high chance of infection in the vicinity of the person who has the infection.

The probability for generating the first infected person, *p_i_*, was fixed at 0.000001. This is the probability level at which 0.24 out of 10,000 people per day get infected by chance. In each scenario, the simulation was carried out by varying the probability *p_x_* of the rapid detection of infected individuals from 1/12, 1/24, 1/48, 1/72, 1/96, 1/120, 1/144, to 1/168, and the probability *p_t_* related to the infection rate from 0.1/24, 0.2/24, 0.3/24, 0.4/24, 0.5/24, 0.6/24, 0.7/24, to 0.8/24. These values of *p_x_* indicate that it takes an average of 12 hours, 24 hours (one day), and 48 hours (two days) for one infected person to be confirmed of infection. And these values of *p_t_* represent 10%, 20%, and 30% chance that an infected person makes those who are connected to him/her infected in a day. Since it is a stochastic calculation, the simulation was performed 100 times under the same parameters to calculate the average results.

**Figure 4.**
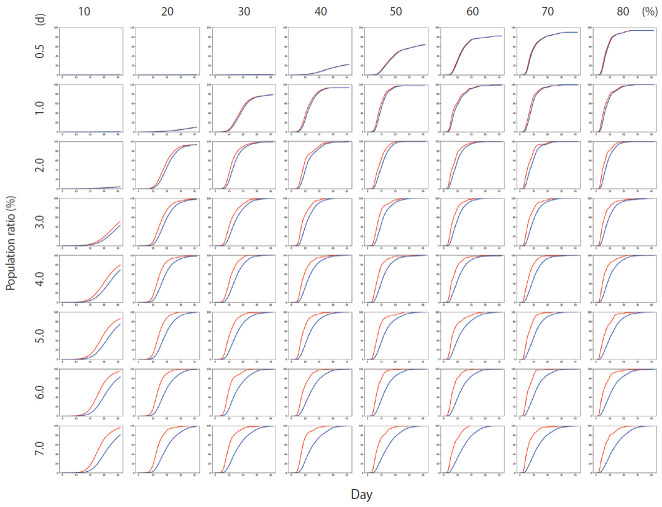
The percentage of the cumulative number of infected people for Scenario 1 (red line) and the percentage of the cumulative number of persons with confirmed infection (blue line). The *y*-axis of each graph represents the percentage of the population and ranges from 0-100%, while the *x*-axis represents time and ranges from 0-40 days. The period between 0.5-7.0 days on each row is the average time required for an infected person to be detected. Each column from 10-80% indicates the infection rate.

**Figure 5.**
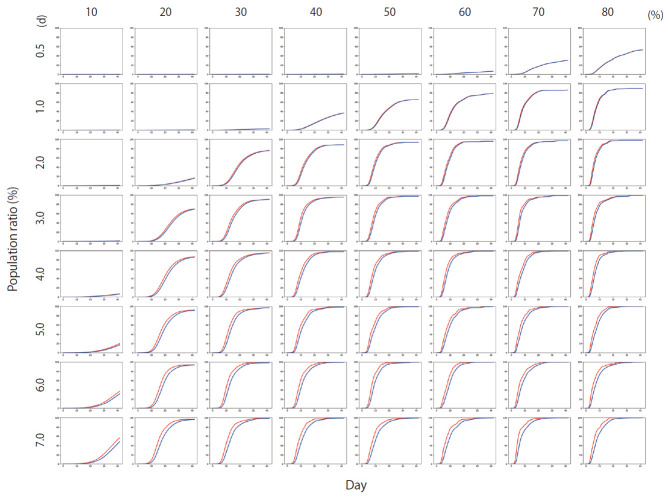
The percentage of the cumulative number of infected people for Scenario 2 (red line) and the percentage of the cumulative number of persons with confirmed infection (blue line). The *y*-axis of each graph represents the percentage of the population and ranges from 0-100%, while the *x*-axis represents time and ranges from 0-40 days. The period between 0.5-7.0 days on each row is the average time required for an infected person to be detected. Each column from 10-80% indicates the infection rate.

**Figure 6.**
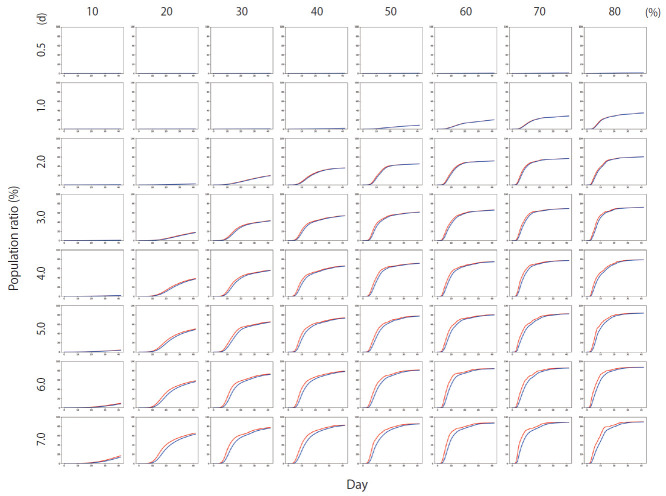
The percentage of the cumulative number of infected people for Scenario 3 (red line) and the percentage of the cumulative number of persons with confirmed infection (blue line). The *y*-axis of each graph represents the percentage of the population and ranges from 0-100%, while the *x*-axis represents time and ranges from 0-40 days. The period between 0.5-7.0 days on each row is the average time required for an infected person to be detected. Each column from 10-80% indicates the infection rate.

Figure 4 shows the results of Scenario 1. Figure 4 shows the trends in the cumulative number of the infected individuals (*I*) and the number of detected infections (*I_x_*) over time under different conditions. A large number of people were infected as the infection rate increased. In addition, if the detection of an infection was delayed, a large number of people were infected. Figure 5 shows the results for Scenario 2. A trend similar to that of Scenario 1 is retained. However, the spread of infection is suppressed in a wider range of conditions. Figure 6 shows the results for Scenario 3. A trend similar to that of Scenarios 1 and 2 is retained, and the spread of infection is suppressed under conditions wider than those of Scenario 2.

**Figure 7.**
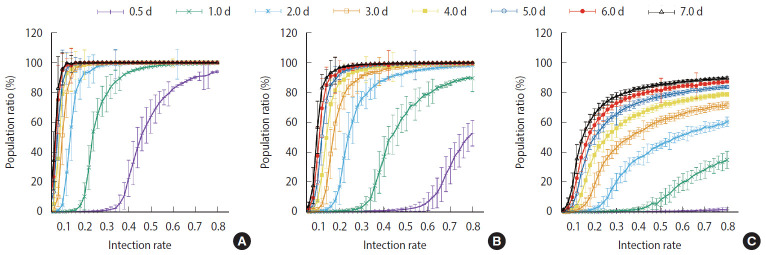
Percentage of the cumulative infected population at the end of the simulation (Day 40) for (A) Scenario 1, (B) Scenario 2, and (C) Scenario 3. Average of 100 simulation results. Error bar is the size of the standard deviation in the results of 100 simulations. The *y*-axis of each graph ranges from 0-100% as the percentage of the cumulative number of infected people compared to total population, and the *x*-axis represents the infection rate.

Figure 7 shows the comparisons of the final cumulative number of infected people at the end of the simulation (Day 40) according to the different parameters. The final cumulative number of infected people decreased overall as the detection time became shorter. In Scenario 1, a relatively large proportion of the population was infected despite a very rapid detection time of 12 hours, when the infection rate was high. In Scenario 3, in which rapid detection of infectors and isolation of everyone around the infector for a certain period of time is combined, the spread of the infection was well suppressed even in situations of a fairly high infection rate.

## DISCUSSION

We proposed an abstract model that could verify the effect of rapid infection detection and subsequent social contact blockage in relation to the spread of COVID-19. Simulations were performed under various conditions for two parameters: the detection time for an infected person and the rate at which the infection spreads. We also looked at the intervention effect according to three different scenarios. The first scenario is to block social contact only for individuals whose infection was detected. The second scenario is to block social contact of the person whose infection is recognized and the only infected person who is directly connected to the detected infector. The third scenario is to block social contact for 14 days, regardless of infection, for all people who are directly connected to the person with confirmed infection.

For the measures taken in Scenario 1, a significant proportion of the population became infected regardless of how quickly the infections was detected when the infection rate was at least 40%. In Scenario 2, the level of infection suppression was higher than that observed in Scenario 1. Still, when the infection rate was at least 60%, the spread of infection was difficult to suppress despite rapid detection of infection. It can be seen that the containment measures of Scenario 3 and the rapid detection of infected persons can be almost completely blocked from infection at any infection rate. Nevertheless, with an infection rate of at least 50%, a significant proportion of the population became infected even in Scenario 3 when the detection of infection took 24 hours on average.

The current policy for controlling the spread of COVID-19 infection in Korea is to isolate those infected as well as anyone who came in direct contact with the infected for 14 days. Also, rapid detection of infected individuals can further suppress the spread of infection, so it is imperative that the quarantine authorities conduct screening tests to identify infected individuals within the shortest time possible. This policy for controlling COVID-19 in Korea has the same objective of optimally implementing the measures assumed in Scenario 3.

Using a simulation based on three scenarios, we have verified that the rapid detection and isolation of infected individuals are critical factors to prevent the spread of infectious diseases. COVID-19 is known to be able to transmit the virus to others even in asymptomatic or mildly symptomatic patients [11-14]. Therefore, there is concern regarding the spread of infection by infected individuals who cannot be identified quickly. However, the results of the simulation demonstrated that rapid identification of infected individuals using epidemiological investigations and contact tracing as well as appropriately isolating those around the infected can greatly suppress the spread of COVID-19.

## SUPPLEMENTARY MATERIALS

Korean version is available at http://www.e-epih.org/.
