# Supplementary material for: Intervention effects in the transmission of COVID-19 depending on the detection rate and extent of isolation
Source: Epidemiol Health. 2020 Jun 23;42:e2020045. doi: 10.4178/epih.e2020045 (PMC7644942; doi:10.4178/epih.e2020045)
Supplement: Supplementary file 1 [file epih-42-e2020045-suppl.docx]

**코로나 19 전파에 있어서 환자 발견 속도와 격리 정도 수준에 따른 중재 효과**

권오규^1^, 손우식^1^, 김진용^2^, 김종헌^3^ ^*^

^1^국가수리과학연구소, 대전, 대한민국

^2^ 인천광역시의료원 감염내과, 인천, 대한민국

^3^ 성균관대학교 의과대학 사회의학교실, 수원, 경기도, 대한민국

한글 초록:

목적(Objectives): 2020년 국내에서 호흡기 감염병인 코로나-19 감염자가 확산되고 있다. 감염병의 확산을 막기 위해서는 감염된 사람을 신속히 발견하여 격리시켜야 하며, 접촉자가 발생하는 것을 조기에 차단하여야 한다. 본 연구는 이러한 조치를 취함으로써 나타나는 감염병 확산의 억제 효과를 수리 모델을 사용하여 검증하고자 하였다.

방법(Methods): 특별한 구조의 네트워크로 연결된 가상의 인구 집단을 대상으로 한 Susceptible-Infectious-Recovery (SIR)모델을 사용하였다. 모델의 감염된 상태($I$)를 감염이 비인지된 상태 $I$와 감염이 인지된 상태 $I_{x}$로 구분한 모형을 적용하였다. $I$ 상태에서 $I_{x}$로 전이하는 확률은 감염자가 발견되는 속도와 동일하며, 본 연구의 네트워크에서는 서로 연결된 사람들만 감염 발생이 가능한 것으로 가정하였다. 또한 본 연구에서는 이러한 사람들간의 연결을 일시적으로 제거함으로써 격리의 효과를 구현하여 평가하고자 하였다.

결과(Results): 시나리오1은 감염이 확인된 본인만 격리되는 경우이며, 시나리오 2는 감염이 확인된 본인과 연결되어 있는 사람 중에서 감염된 사람을 신속히 감염자로 인지하여 함께 격리되는 경우이다. 시나리오 3은 감염이 확인된 당사자와 연결된 모든 사람을 격리하는 경우로 하여 시뮬레이션을 시행하였다. 시나리오 3의 경우는 상대적으로 느린 확진 속도에서와 비교적 높은 감염율에서도 효과적으로 감염병을 억제하는 결과를 얻을 수 있었다.

결론(Conclusions): 감염병이 유행하는 시기에는 감염자의 신속한 발견이 도움이 된다. 또한 본인 및 주변 사람들을 함께 관리하는 것이 감염병 확산의 억제에 상당한 도움이 된다는 것을 시뮬레이션 평가를 통해 정량적으로 제시할 수 있었다.

핵심 단어: COVID-19, 행위자 기반 모형, 비약물적 중재, 사회 연결망, 격리

Key words: COVID-19, Agent-Based Model, non-pharmaceutical intervention, social network, isolation

**서론**

2019년 11월 중국에서 처음 보고되었으며, 코로나바이러스감염증-19로 이름 붙여진 새로운 바이러스 감염병에 대해 2020년 3월 11일 세계보건기구에서는 대유행(pandemic)을 선포하였다 [1]. 새로운 감염병이 전세계적으로 유행하는 시기에는 치료제나 백신을 쉽게 얻을 수 없기 때문에 비약물적 중재(non-pharmaceutical intervention)를 시행하여 감염병 유행으로 인한 환자 발생과 관련된 최고 정점을 크기를 낮추고, 한 국가에서의 보건의료 역량 수준을 넘어서지 않도록 체계를 유지하는 “완화 전략”을 시행하는 것이 거의 유일한 대응방법으로 알려져 있다 [2]. 비약제적 중재 중에 가장 직관적이고 효과적인 것으로 많이 알려진 방법은 “감염된 사람을 가능한 빨리 발견하여 격리하는 것”이다 [3,4]. 본 연구에서는 COVID-19를 얼마나 빨리 인지하고 격리를 하는 것이 감염병 전파의 차단에 도움이 되는지를 수리 모델을 통해 알아보고자 하였다.

**방법 (Methods)**

1. 모형(Model)

인구 1만명이 특정한 네트워크로 연결되어 있는 가상의 인구 집단을 가정하였다. 모든 사람들은 네트워크 상에서 연결 선으로 연결되어 있는 사람과만 접촉할 수 있다고 가정하였다. 각 사람은 평균 10명의 다른 사람들과 연결되어 있다. 또한 모든 연결들 중에서 10%가 무작위적인 먼 거리의 연결을 갖는 Watts와 Strogatz가 제안한 좁은 세상 네트워크를 도입하였다 (Figure 1) [5]. 사람들간의 연결 구조는 시간에 따라 변하지 않는다고 가정하였다. 점과 선의 집합인 네트워크에서 점은 개별 인간으로 선은 인간 사이의 감염 통로로 비유하여 감염병 확산을 계산하는 모형은 기존에 많이 연구되어왔다 [6,7,8]. 우리는 질병 모델로는 가장 기초적인 Susceptible-Infectious-Recovery (SIR) 모델을 사용했다 [9,10]. 다만 감염 상태인 $I$를 감염된 상태로 인지하지 못했다면 $I$ 상태 그대로 두고, 감염된 상태를 인지하였다면 $I_{x}$ 상태로 구분하였다 (Figure 2). 감염이 되었을 때 즉시 감염을 인지할 수 없는 것이 현실이다. 또한 감염을 인지하지 못하는 경우에는 일상적인 활동을 지속하면서 다른 사람들에게 감염을 전파시키게 된다. 한편, 감염이 인지된 감염자는 병원에서 치료를 받게 되면 더 이상 감염을 전파하지 않게 된다. 따라서 감염이 되었지만 감염이 인지된 $I_{x}$ 상태가 되면 연결되어 있는 주변 사람들에게 절대로 감염을 전파하지 않는다고 가정하였다. 감염이 인지되지 않은 사람만이 그와 연결되어 있는 사람에게만 특정한 확률($p_{t}$)로 감염 시킬 수 있다. 감염이 된 상태($I$)에서 그 감염이 인지된 상태($I_{x}$)로 변할 수 있는 확률 $p_{x}$가 있다 (Figure 3). $I$ 상태와 $I_{x}$ 상태의 시간 길이를 합하면 총 3주, 즉 21일이 되도록 하였다. 따라서 $p_{x}$ 값이 충분히 작다면 3 주간 $I$ 상태로 머물다가 Recovery ($R$) 상태가 되는 경우도 나타날 수 있다. 최초의 감염자를 발생시키기 위한 확률 $p_{i}$가 있다. 이는 자연 발생적인 감염 혹은 대상 인구 집단의 외부에서 감염되어올 가능성이며 이 값은 상당히 작은 크기로 설정하였다. 모형의 파라미터는 이렇게 $p_{t}$, $p_{x}$, $p_{i}$세 개이다.

시간에 따라 한 개인의 질병 상태가 변화하는 확률적 과정은 그림3과 같으며 시간 간격은 한 시간으로 정하여 계산하였다. 시간 $t$ 에서 Susceptible ($S$) 상태인 사람은 $p_{i}$의 확률로 시간$t+1$ 에 $I$ 상태로 변할 수 있다. 만약 시간 $t$ 에서 $S$ 상태인 사람이 자신과 연결되어 있는 주변 사람 중 감염자가 $n$ 명이 있다면 ${n\times p}_{t}$의 확률이 더해져 시간$t+1$ 에서 $I$ 상태로 변한다. 시간 $t$ 에 $I$ 상태인 사람은 $p_{x}$ 의 확률로 시간$t+1$에서 감염이 인지된 상태 즉 $I_{x}$ 상태로 변하거나 ${1-p}_{x}$의 확률로 $I$ 상태 그대로 남아있다. 시간 $t$ 에 $I$ 상태가 된 사람은 무조건 시간 $t+24\times21$(3주)에 Recovery ($R$) 상태가 된다. 그리고 한 번 $R$ 상태가 된 사람은 이후 계속 $R$ 상태를 유지한다.

**결과(Results**)

신속한 감염자 발견과 격리의 효과를 검증하기 위해 세 가지 시나리오를 적용하여 시뮬레이션을 하였다. 첫번째 시나리오는 감염이 인지된 당사자만 사회적 연결이 차단되는 경우이다. 두번째 시나리오는 감염이 인지된 당사자와 직접 연결되어 있는 사람 중 감염이 확인되면 즉시 인지된 감염자로 바꾸어 사회적 연결이 차단되는 경우이다. 감염자와 접촉한 사람들에 대한 역학 조사를 통하여 발견된 감염자에 대해서는 신속히 감염 여부가 확인되는 상황을 묘사한 것이다. 세번째 시나리오는 감염이 인지된 사람과 직접 연결되어 있는 사람 중 감염이 확인되면 즉시 인지된 감염자로 바꾸고 또한 감염되지 않았다고 하더라도 무조건 14일간 한시적으로 연결을 차단하는 것이다. 감염이 발생한 사람의 근처에는 상대적으로 높은 감염의 가능성이 있을 수 있기 때문에 비감염자도 사회적 접촉을 최소화하는 것이다.

최초의 감염자를 발생시키기 위한 확률 $p_{i}$는 0.000001로 고정하였다. 이 확률은 하루에 만명 중 0.24명의 감염자가 우연히 발생하는 수준이다. 각 시나리오마다 감염자의 신속한 발견과 관련된 확률인 $p_{x}$를 1/12, 1/24, 1/48, 1/72, 1/96, 1/120, 1/144, 1/168인 감염율과 관련된 확률인 $p_{t}$를 0.1/24, 0.2/24, 0.3/24, 0.4/24, 0.5/24, 0.6/24, 0.7/24, 0.8/24로 변화를 주어 시뮬레이션하였다. 감염이 인지되기까지 평균 12시간, 24시간(하루), 48시간(이틀)으로 점점 오래 걸리는 상황, 즉, 감염자가 24시간 이내에 연결되어 있는 사람들을 감염자로 만들 수 있는 확률이 10%, 20%, 30%로 커지는 상황에 대한 확률값들이다. 확률적 계산이므로 동일한 파라미터 조건에서 100번씩 시뮬레이션을 실행하여 그 평균 결과를 산출하였다.

그림 4는 시나리오 1에 대한 결과이다. 그림 4는 여러 조건하에서 누적 감염자($I$) 수와 인지된 감염자($I_{x}$) 수의 시간에 따른 추이를 보여주고 있다. 감염율이 커질 수록 많은 수의 사람들이 감염됨을 확인할 수 있다. 또한 감염이 인지되는 시간이 늦어질수록 많은 수의 사람들이 감염됨을 확인할 수 있다. 그림 5는 시나리오 2에 대한 결과이다. 시나리오 2와 비슷한 경향은 유지가 된다. 하지만 감염의 확산이 더욱 넓은 조건에서 억제되는 것을 확인할 수 있다. 그림 6은 시나리오 3에 대한 결과이다. 시나리오 1 및 2와 비슷한 경향성은 유지되며, 시나리오 2보다 더욱 넓은 조건에서 감염의 확산이 억제되는 것을 확인할 수 있다.

그림 7은 시뮬레이션 계산의 종료 시점인 40일에서의 최종 누적 감염자 수를 파라미터에 따라 비교한 것이다. 감염이 인지되는 시간이 짧아 질 수록 최종 누적 감염자 수는 전체적으로 줄어든다. 시나리오 1의 경우는 12시간이라는 아주 신속한 감염 인지의 상황에서도 감염율이 크면 적지 않은 인구가 감염됨을 확인할 수 있다. 신속한 감염자 확인과 그에 따라 주변 모두를 일정 기간 격리 조치하는 경우인 시나리오 3의 경우는 상당히 큰 감염률의 상황에서도 훌륭하게 감염의 확산이 억제됨을 보여주고 있다.

**토론(Discussion)**

우리는 코로나-19 확산과 관련하여 신속한 감염자 발견과 그에 따른 주변 사람들의 사회적 접촉 차단의 효과를 검증할 수 있는 추상적인 모형을 제안하였다. 감염자의 감염이 인지되기까지 소요되는 시간과 감염이 전파되는 속도 이 두가지의 파라미터 대한 다양한 조건하에서 시뮬레이션을 수행하였다. 또한 접촉자 관리의 세 가지 시나리오에 대하여 그 효과를 살펴보았다. 첫 번째 시나리오는 감염이 인지된 당사자만 사회적 접촉을 차단하는 것이다. 두 번째 시나리오는 감염이 인지된 사람과 그와 직접적으로 연결되어 있는 사람 중 감염자만 감염 인지자로 바꾸고 사회적 접촉을 차단하는 것이다. 세 번째 시나리오는 감염이 인지된 사람과 그와 직접적으로 연결되어 있는 비감염자도 14일간 사회적 접촉을 차단하는 격리를 실행하는 것이다.

시나리오 1의 조치에서는 아무리 빠르게 감염자가 발견된다 하더라도 질병의 감염율이 40% 이상이면 상당한 수준의 인구가 감염되는 결과가 나타났다. 시나리오 2의 조치에서는 시나리오 1의 조치보다는 감염을 억제하는 수준이 높아짐을 확인할 수 있었다. 그렇지만 이 경우에도 아무리 신속한 감염자 발견이 이루어지더라도 60% 이상의 감염율에 대해서는 감염의 확산을 억제하기 어려움을 확인하였다. 시나리오 3 조치와 신속한 감염자 발견은 어떠한 감염율의 상황에서도 거의 완벽하게 감염이 차단될 수 있음을 볼 수 있다. 그렇더라도 감염의 인지가 평균 하루 정도 걸리는 상황과 감염율이 50% 이상인 상황에서는 시나리오 3의 조치 상황에서도 상당한 인구가 감염되는 상황이 나타났다.

현재 국내에서 시행하고 있는 코로나19 감염병에 대한 확산 통제 정책은, 감염된 사람뿐만 아니라 그와 직접적으로 접촉한 모든 사람들을 14일간 자가 격리하는 조치를 취하고 있다. 또한 감염자의 발견은 빠르면 빠를수록 감염의 확산을 더욱 억제할 수 있기 때문에, 방역당국은 최대한 빠른 시간 내에 감염자를 찾아내기 위해 선별 검사를 시행하고 있다. 이러한 국내에서의 코로나 19 감염병 확산 통제 정책은 우리가 본 연구에서 제시한 시나리오 3에서 가정한 조치를 최적으로 이행하고자 하는 것과 목표점이 같다고 할 수 있다.

우리는 3가지 시나리오에 근거한 시뮬레이션을 통하여 신속한 감염자의 발견과 격리가 감염병의 확산을 통제하는데 중요한 요소임을 확인하였다. 코로나-19는 무증상 상태나 미약한 임상양상을 나타내고 있는 상태에서도 바이러스를 타인에게 전파할 수 있는 것으로 알려져 있다 [11-14]. 따라서 신속히 발견되기 어려운 감염자에 의해 감염이 확산될 수 있는 우려가 있다. 하지만 역학조사와 접촉자 추적시스템을 활용하여 가능한 신속하게 감염자를 찾아내고, 그 감염자의 주변 모든 사람들을 적절히 격리 조치하는 것은 코로나-19 감염 확산의 억제에 큰 도움을 줄 수 있음을 시뮬레이션 결과는 보여주고 있다.

**Acknowledgement**

We thanks to all of the persons who were struggling in healthcare fields to overcome the COVID-19 outbreak. This study was performed under the research project named ‘Research and Development on Integrated Surveillance System for Early Warning of Infectious Diseases (RISEWIDs)’. The investigators of this project were Jaiyong Kim (Yonsei University), Sunworl Kim (National Medical Center), Eui Jung Kwon (National Health Insurance Review and Assessment Service), Dong Wook Kim (National Health Insurance Service), Moran Ki(National Cancer Center), Hyunjin Son (Pusan National University Hospital), Jong-Hun Kim (Sungkyunkwan University), Jin Yong Kim (Incheon Medical Center), Heeyoung Lee (Seoul National University Bundang Hospital), Boyoung Park (Hanyang University), Woo-Sik Son (National Institute for Mathematical Sciences), Jungsoon Choi (Hanyang University), Sunhwa Choi (National Cancer Center), Okyu Kwon (National Institute for Mathematical Sciences), Hyojung Lee (National Institute for Mathematical Sciences), Jong-Hoon Kim (International Vaccine Institute), Heecheon Kim (MISO INFO TECH), and Bo Youl Choi (Hanyang University). This project was supported by Government-wide R&D Fund Project for Infectious Disease Research (GFID), Republic of Korea (Grant No. HG18C0088). This project was also supported by the National Institute for Mathematical Sciences founded by the Ministry of Science, ICT & Future Planning (B20900000)

**Conflict of interest**

The authors have no conflicts of interest to declare for this study.

**REFERENCES**

1. Mahase E. Covid-19: WHO declares pandemic because of "alarming levels" of spread, severity, and inaction. BMJ 2020;368:m1036.

2. Ferguson N, Laydon D, Nedjati Gilani G, Imai N, Ainslie K, Baguelin M, et al. Report 9: Impact of non-pharmaceutical interventions (NPIs) to reduce COVID19 mortality and healthcare demand. 2020.

3. Hellewell J, Abbott S, Gimma A, Bosse NI, Jarvis CI, Russell TW, et al. Feasibility of controlling COVID-19 outbreaks by isolation of cases and contacts. Lancet Glob Health 2020;8:e488-e496.

4. Niu Y, Xu F. Deciphering the power of isolation in controlling COVID-19 outbreaks. Lancet Glob Health 2020;8:e452-e453.

5. Watts DJ, Strogatz SH. Collective dynamics of ‘small-world’networks. Nature 1998;393:440.

6. A networked SIR model [cited 2020 May 30] Available from http://systems-sciences.uni-graz.at/etextbook/networks/sirnetwork.html

7. Keeling MJ, Eames KT Networks and epidemic model. Journal of the Royal Society Interface 2005; 2:295-307.

8. Jo HH, Baek SK, Moon HT Immunization dynamics on a two-layer network model. Physica A: Statistical Mechanics and it’s applications 2006; 361:534-542.

9. Kermack WO, McKendrick AG. A contribution to the mathematical theory of epidemics. Proceedings of the royal society of london. Series A, Containing papers of a mathematical and physical character 1927;115:700-721.

10. Anderson RM, May RM. Population biology of infectious diseases: Part I. Nature 1979; 280:361-367.

11. Bai Y, Yao L, Wei T, Tian F, Jin DY, Chen L, et al. Presumed Asymptomatic Carrier Transmission of COVID-19. JAMA 2020.

12. Li C, Ji F, Wang L, Wang L, Hao J, Dai M, et al. Asymptomatic and Human-to-Human Transmission of SARS-CoV-2 in a 2-Family Cluster, Xuzhou, China. Emerg Infect Dis 2020; 26.

13. Pan X, Chen D, Xia Y, Wu X, Li T, Ou X, et al. Asymptomatic cases in a family cluster with SARS-CoV-2 infection. Lancet Infect Dis 2020; 20:410-411.

14. Tong ZD, Tang A, Li KF, Li P, Wang HL, Yi JP, et al. Potential Presymptomatic Transmission of SARS-CoV-2, Zhejiang Province, China, 2020. Emerg Infect Dis 2020; 26.

**Figure legend**

**Figure 1**. Watts와 Strogatz가 제안한 좁은 세상 네트워크의 개념도.

각각의 점은 사람을 의미하며, 점과 점 사이를 이은 선은 그 사람들 사이에 연결 관계가 있음을 의미함. 본 그림은 시각적 편의를 위해 25명이 연결되어 있는 예를 보여주고 있음

**Figure 2**. 한 사람의 시간에 따른 사회적 연결 상태의 변화 과정.

감수성 상태(S)에서는 주변의 감염된 이웃 사람들로부터 감염이 될 수 있음. 감염이 된 상태(I)는 ∆T의 시간 동안 연결되어 있는 주변 사람에게 감염을 유발할 수 있음. I 상태가 되고 ∆T 시간이 지나면 감염을 인지하여 Ix 상태가 됨. Ix 상태에서는 사회적 연결이 차단되어 주변에 감염을 유발할 수 없음. 감염으로부터 회복된 R 상태가 되면 사회적 연결이 회복되어도 감염을 전파

**Figure 3**. 시간의 변화에 따른 SIR 모델의 확률적 변화

t 시간에서의 상태가 t+1 시간에서의 상태로 확률적으로 변함. 최초 I 상태가 시작된 이후로 I 상태로 남아있건 Ix 상태로 변하였건 21일(3주)이 지나면 무조건 R 상태가 됨. $p_{i}$는 1차 감염자의 발생 확률임. $np_{t}$는 $S$ 상태인 자와 연결되어 있는 $n$ 명의 $I$ 상태의 감염자가 전염 발생시키는 확률임. $p_{x}$는 $I$ 상태의 감염자가 확진 판정을 받게 되는 확률임.

**Figure 4**. 시나리오 1에 대한 누적 감염된 인구의 비율 (빨간 선) 및 누적 감염이 확인되어 인지된 인구 비율 (파란 선).

각 그래프의 y축은 인구 비율로 0%-100%의 범위를 갖고, x축은 시간(일 단위)으로 0-40일의 범위를 갖음. 각 행에 표시된 0.5day부터 7.0day는 감염자가 확진자가 되는 평균 기간임. 각 열에 표시된 10%에서 80%는 감염율임. 각 그래프의 y축은 인구 비율로 0%-100%의 범위를 갖고, x축은 시간(일 단위)으로 0-40일의 범위를 갖음.

**Figure 5**. 시나리오 2에서 누적 감염된 인구의 비율 (빨간 선) 및 누적 감염이 확인되어 인지된 인구 비율 (파란 선).

각 그래프의 y축은 인구 비율로 0%-100%의 범위를 갖고, x축은 시간(일 단위)으로 0-40일의 범위를 갖음. 각 행에 표시된 0.5day부터 7.0day는 감염자가 확진자가 되는 평균 기간임. 각 열에 표시된 10%에서 80%는 감염율임. 각 그래프의 y축은 인구 비율로 0%-100%의 범위를 갖고, x축은 시간(일 단위)으로 0-40일의 범위를 갖음.

**Figure 6**. 시나리오 3에서 누적 감염된 인구의 비율 (빨간 선) 및 누적 감염이 확인되어 인지된 인구 비율 (파란 선).

각 그래프의 y축은 인구 비율로 0%-100%의 범위를 갖고, x축은 시간(일 단위)으로 0-40일의 범위를 갖음. 각 행에 표시된 0.5day부터 7.0day는 감염자가 확진자가 되는 평균 기간임. 각 열에 표시된 10%에서 80%는 감염율임. 각 그래프의 y축은 인구 비율로 0%-100%의 범위를 갖고, x축은 시간(일 단위)으로 0-40일의 범위를 갖음.

**Figure 7**. 시뮬레이션 종료 시점인 40일에서의 누적 감염된 인구의 비율.

100번 시뮬레이션 결과의 평균값. 오차막대기는 100번 시뮬레이션 결과의 표준편차 크기. (a) 시나리오 1, (b) 시나리오 2, (c) 시나리오 3.

각 그래프의 y축은 전체 인구대비 누적 감염자 수의 비율로 0%-100%의 범위를 갖고, x축은 감염율임.
